# Azimuthally dependent absorption and gain in an atomic system with spontaneously generated coherence controlled by an optical vortex field

**DOI:** 10.1038/s41598-025-96197-y

**Published:** 2025-04-15

**Authors:** Viačeslav Kudriašov, Teodora Kirova, Seyyed Hossein Asadpour, Hamid R. Hamedi

**Affiliations:** 1 Institute of Theoretical Physics and Astronomy, Vilnius University, 10257 Vilnius, Lithuania; 2 Faculty of Science and Technology, Institute of Atomic Physics and Spectroscopy, University of Latvia, Riga, 1004 Latvia; 3 School of Quantum Physics and Matter, Institute for Research in Fundamental Sciences, IPM, Tehran, 19538-33511 Iran

**Keywords:** Optics and photonics, Atomic and molecular physics, Optical physics, Quantum physics

## Abstract

This paper examines spatially dependent absorption and gain of a weak probe field in a 3-level atomic $\Lambda$-system with spontaneously generated coherence and incoherent pump, using an optical vortex beam as the control field. The inhomogeneous phase distribution between the probe and vortex fields modulates the transverse distribution of the system’s optical response. A flexible control over the azimuthal modulation in the transverse plane can be achieved by changing such parameters as the orbital angular momentum and the optical transition detuning. The vortex parameters can also be used to affect the population distribution of the atomic system’s upper state. This method for achieving precise control over the optical response characteristics contributes to advances in optical information and communication, quantum devices and sensing.

## Introduction

The interaction of strong coherent fields with multilevel atomic systems leads to dynamically induced coherence, which results in novel coherent radiation sources without the need for population inversion. The latter represents the well-known concepts of amplification or lasing without inversion (AWI, LWI), which have been both theoretically studied and experimentally realized in a variety of schemes in quantum optics^1–8^. In the case of a system with closely lying (near-degenerate) states, the laser-matter interaction gives rise to a new type of coherence, a spontaneously generated coherence (SGC)^9^, which causes quantitative changes in the absorption/dispersion spectra, making the line shapes dependent on the relative phase of the applied fields^10^. For SGC to occur, two essential criteria must be satisfied: (i) the frequency separation between the ground-state levels must be comparable to or smaller than the excited-state decay rate, ensuring near-degeneracy, and (ii) the dipole transition moments associated with these levels must be non-orthogonal. Wu and Gao^11^ showed theoretically that, by introducing incoherent pumping in a $\Lambda$ system, one can achieve phase control of the inversionless gain by using the SGC process. The mechanism of phase control via SGC has also been demonstrated in various *V*-^12^, ladder-^13^, double-$\Lambda$-^14,15^, *N*-^15,16^, as well as inverted *Y*-systems^17^. Other interesting applications include Kerr nonlinearity^18,19^, electromagnetically induced grating^20,21^, and atom-photon entanglement^22^.

A range of intriguing effects in quantum optics also arises when utilizing the additional degrees of freedom provided by the orbital angular momentum (OAM) of light. Light beams carrying OAM, e.g. optical vortex beams, have ring-shape intensity profiles, as well as helical shape wavefronts^23,24^. Information can be encoded in the different values of OAM, creating multiple channels for information transmission and thus, increasing the capacity of optical technologies, such as data transmission, optical communication^25,26^ and quantum information^27^. Various applications originating from the atom interactions with optical vortices involve optical tweezers^28^, light-induced-torque^29,30^, implementation of an atomic compass^31^, OAM-based four-wave mixing^32,33^, as well as entanglement of OAM states of photon pairs^34,35^. Vortex beams also yield novel prospects for the manipulation of the optical information via slow light storage and retrieval^36–39^. Despite their many applications, so far, the optical vortices have rarely been utilized in the atomic SGC and AWI schemes^40^, while we expect that such implementations will give possibilities for additional control, due to the extra degrees of freedom carried by the OAM.

To this end, here we study the spatial dependence of absorption and gain properties of a weak probe field in a 3-level $\Lambda$-system exhibiting SGC, where the control field is represented by a vortex beam. In this setup, the relative phase between the plane probe field and the vortex control field is spatially inhomogeneous, leading to the modulation in the transverse distributions of the dispersive and absorptive properties in the system. Our main goal is to focus on the amplitude and phase dependent absorption effects across the transverse profile of the applied probe field. In what follows we analyze and discuss the modulated transverse patterns for the certain values of the relevant optical parameters and their combinations. In particular, we explore the effect of OAM number, field detunings, dipole angle, vortex strength, and incoherent pump rate. Our results demonstrate that such a setup, involving amplitude and phase structured control field, provides high flexibility in handling and patterning the optical response characteristics, and thus may have potential applications in optical information and communication, advancing novel quantum devices and sensing.

**Fig. 1:**
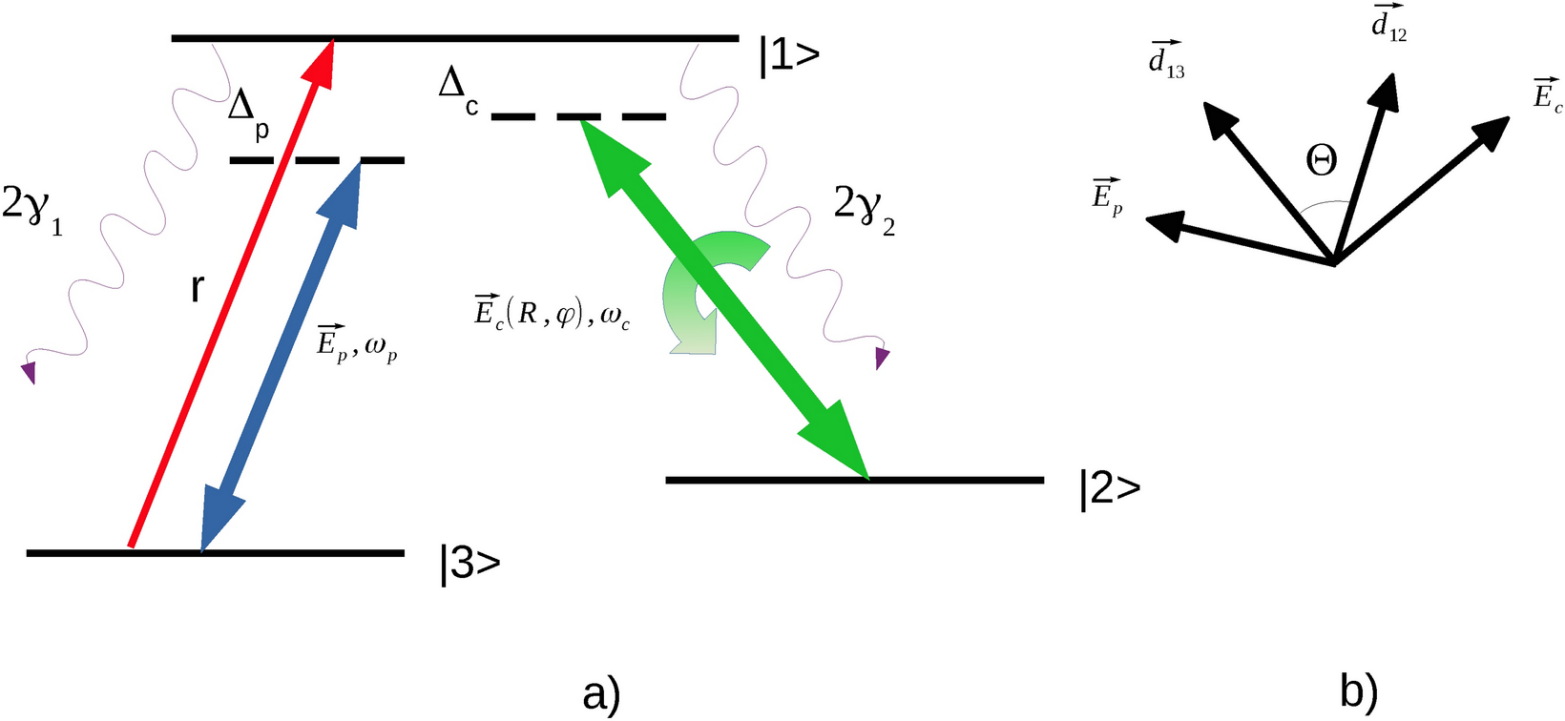
(a) Schematic representation of the 3-level $\Lambda$-scheme; (b) Polarization directions of electric fields and dipoles.

The paper is organized as follows. First, we introduce a theoretical model for describing the 3-level $\Lambda$-scheme under consideration, as well as the basic set of equations, by solving analytically the density-matrix equations of motion. The numerical study of absorption (gain) and state population depending on different system parameters (OAM, field detunings, field strengths, incoherent pump rate), along with the corresponding discussions, are presented in the subsequent section. We conclude the paper by summarizing our findings and proposing possible experimental implementations in suitable quantum systems.

## Model and equations

The optical system under study (depicted in Fig. 1) is a three-level $\Lambda$-scheme consisting of an upper energy level $|1\rangle$ and two lower near-degenerate energy levels $|2\rangle$ and $|3\rangle$^11^. The system is driven by three optical fields: a weak probe field $\vec {E_p}$, a strong control field $\vec {E_c}$, as well as an incoherent pump *r*. The specifics of this setup is a quantum interference (QI) between the two spontaneous emission channels $|1\rangle$- $|2\rangle$ and $|1\rangle$-$|3\rangle$. In the case when $|2\rangle$ and $|3\rangle$ levels are located sufficiently close (nearly degenerate), the QI leads to the spontaneously generated coherence, further enhanced by the presence of an incoherent pump in this setup. As noted in^11^, the role of the incoherent pump is crucial for the SGC in the case of a weak probe beam. In this arrangement, the atomic population is initially distributed across all the three levels, which is different from the typical electromagnetically induced transparency^41,42^ cases, where the atom population starts from the ground state. Differently from the previous works, here we explore the above setup in the conditions of the spatially inhomogeneous optical excitation (both in the amplitude and phase) due to the control field being a vortex beam carrying OAM.

Utilizing the density matrix approach^43^ and following^11^, the equations of motion for the density matrix elements of the 3-level $\Lambda$- atomic system can be written as follows

1 $$\begin{aligned} \frac{\partial \rho _{22}}{\partial t}= & 2\gamma _{2}\rho _{11}+iG_{c}^{*}\rho _{12}-iG_{c}\rho _{21}, \end{aligned}$$

2 $$\begin{aligned} \frac{\partial \rho _{33}}{\partial t}= & 2\gamma _{1}\rho _{11}-2r\rho _{33}+iG_{p}^{*}\rho _{13}-iG_{p}\rho _{31}, \end{aligned}$$

3 $$\begin{aligned} \frac{\partial \rho _{12}}{\partial t}= & -(\gamma _{1}+\gamma _{2}+i\Delta _{c})\rho _{12}+iG_{p}\rho _{32}-iG_{c}(\rho _{11}-\rho _{22}), \end{aligned}$$

4 $$\begin{aligned} \frac{\partial \rho _{13}}{\partial t}= & -(\gamma _{1}+\gamma _{2}+r+i\Delta _{p})\rho _{13}+iG_{c}\rho _{23}-iG_{p}(\rho _{11}-\rho _{23}), \end{aligned}$$

5 $$\begin{aligned} \frac{\partial \rho _{23}}{\partial t}= & -(r+i\Delta _{p}-i\Delta _{c})\rho _{23}+2\sqrt{\gamma _{1}\gamma _{2}}\cos \varTheta \eta \rho _{11}-iG_{p}\rho _{21}+iG_{c}^{*}\rho _{13}. \end{aligned}$$

Here $\gamma _{1}$ and $\gamma _{2}$ denote the spontaneous emission rates from the upper level $|1\rangle$ to levels $|3\rangle$ and $|2\rangle$, respectively, while $\Delta _{p}$ and $\Delta _{c}$ are the detunings of the probe and control fields. The angle between the electric dipole moments $\vec {d_{12}}$ and $\vec {d_{13}}$ is represented by $\varTheta$; parameter $\eta$ accounts for the SGC effect taking value $\eta =1$ when SGC is present, or $\eta =0$ otherwise. Rabi frequencies of the probe and control beams are represented by $G_p = (\vec {d_{13}} \cdot \vec {E_p})/\hbar = \Omega _p\sin \varTheta$ and $\quad G_c = (\vec {d_{12}} \cdot \vec {E_c})/\hbar = \Omega _c\sin \varTheta$, respectively.

The QI effect between the spontaneous emission channels is described by the term $p=2\sqrt{\gamma _{1}\gamma _{2}}\cos \varTheta \eta$. This effect takes place when the upper state decays to the two closely spaced lower energy levels. As noted in^11^, for this to happen, the involved electric dipoles must be non-orthogonal (e.g. $\varTheta \ne \pi /2$), with the maximum interference occurring when they are nearly parallel or anti-parallel. However, the perfect QI ($\varTheta =0$) is not possible here, as in such a case both Rabi frequencies for the laser fields coupling each transition would reduce to zero.

To analyze the system’s response in the weak probe field limit, we apply a perturbative expansion of the density matrix elements in terms of the small parameter $G_p$. Since the probe field is weak compared to the control field, we expand the density matrix elements as follows

6 $$\begin{aligned} \rho _{ij} = \rho _{ij}^{(0)} + \rho _{ij}^{(1)} + \mathcal {O}(G_p^2), \end{aligned}$$

where $\rho _{ij}^{(0)}$ represents the zero-order solution (independent of $G_p$), and $\rho _{ij}^{(1)}$ corresponds to the first-order correction due to the weak probe field. The higher-order terms $\mathcal {O}(G_p^2)$ are neglected since the probe field is assumed to have negligible intensity. Substituting this expansion into the density matrix equations and collecting terms of different orders in $G_p$, we first solve for $\rho _{ij}^{(0)}$ by setting $G_p = 0$. This corresponds to the steady-state population distribution in the absence of the probe field, determined only by the control field and incoherent pump. The zero-order solutions become

7 $$\begin{aligned} \rho _{11}^{(0)}= & \frac{r(\gamma _{1}+\gamma _{2})|G_{c}|^{2}}{D}, \end{aligned}$$

8 $$\begin{aligned} \rho _{22}^{(0)}= & \frac{r(\gamma _{1}+\gamma _{2})|G_{c}|^{2}+r\gamma _{2}[(\gamma _{1}+\gamma _{2})^{2}+\Delta _{c}^{2}]}{D}, \end{aligned}$$

9 $$\begin{aligned} \rho _{33}^{(0)}= & \frac{\gamma _{1}(\gamma _{1}+\gamma _{2})|G_{c}|^{2}}{D}, \end{aligned}$$

10 $$\begin{aligned} \rho _{12}^{(0)}= & \frac{\gamma _{2}rG_{c}(\Delta _{c}+i\gamma _{1}+i\gamma _{2})}{D}, \end{aligned}$$

11 $$\begin{aligned} \rho _{13}^{(0)}= & \frac{ipG_{c}\rho _{11}^{(0)}}{(\gamma _{1}+\gamma _{2}+r)(r-i\Delta _{c})+|G_{c}|^{2}}, \end{aligned}$$

12 $$\begin{aligned} \rho _{23}^{(0)}= & \frac{p(\gamma _{1}+\gamma _{2}+r)\rho _{11}^{(0)}}{(\gamma _{1}+\gamma _{2}+r)(r-i\Delta _{c})+|G_{c}|^{2}}, \end{aligned}$$

13 $$\begin{aligned} D= & (\gamma _{1}+\gamma _{2})(2r+\gamma _{1})|G_{c}|^{2}+r\gamma _{2}[(\gamma _{1}+\gamma _{2})^{2}+\Delta _{c}^{2}]. \end{aligned}$$

Then, we solve for $\rho _{ij}^{(1)}$ by keeping terms linear in $G_p$, which describes the system’s linear response to the probe field. The steady-state solutions for $\rho _{11}^{(1)}$ and $\rho _{31}^{(1)}$ are then derived as follows

14 $$\begin{aligned} \rho _{11}^{(1)}= & \rho _{11}^{(0)}+\frac{iG_{p}\{\Delta _{c}r(G_{c}\rho _{23}^{(0)}-G_{c}^{*}\rho _{32}^{(0)})+(\gamma _{1}+\gamma _{2})[|G_{c}|^{2}(\rho _{31}^{(0)}-\rho _{13}^{(0)})-ir(G_{c}\rho _{23}^{(0)}+G_{c}^{*}\rho _{32}^{(0)})]\}}{2D}, \end{aligned}$$

15 $$\begin{aligned} \rho _{31}^{(1)}= & \frac{iG_{p}[(r-i\Delta _{p}+i\Delta _{c})(\rho _{11}^{(0)}-\rho _{33}^{(0)})-iG_{c}^{*}\rho _{12}^{(0)}]-ip^{*}G_{c}\rho _{11}^{(1)}}{(\gamma _{1}+\gamma _{2}+r-i\Delta _{p})(r+i\Delta _{c}-i\Delta _{p})+|G_{c}|^{2}}. \end{aligned}$$

The Rabi frequency of the control vortex beam can be represented in polar coordinates *R* and $\varphi$ as:

16 $$\begin{aligned} \varOmega _{c}=\varOmega _{c}(R,\varphi )=|\varOmega _{c}(R,\varphi )|e^{il\varphi }=\varOmega _{c0}\left( \frac{R}{w}\right) ^{|l|}e^{-R^{2}/w^{2}}e^{il\varphi }, \end{aligned}$$

where *w* is the beam waist, $\varOmega _{c0}$ is the amplitude of the vortex beam, and *l* indicates the OAM number. The Rabi frequency of the probe field can be denoted as $\varOmega _{p}=\varOmega _{p0}$ which is a constant.

## Results

In what follows we present the transverse distributions of the absorption (gain) as well as the upper level’s population, based on the numerical analysis of Eqs. (14) and (15) as dependent on the set of different parameters in the system.

We start with the parameters explicitly affecting the azimuthal dependency (the OAM and detunings), and then turn to those which have weaker azimuthal influence (the angle between the dipoles, the control field strength, and the incoherent pump rate). In our numerical calculations we use the following basic set of parameter values: $r=0.1 \gamma$, $\varOmega _{p0}=0.1 \gamma$, $\varOmega _{c0}=10 \gamma$, $l=1$, $\Delta _{p}=0$, $\Delta _{c}=0$, $\eta =1$, $\varTheta =\pi /4$. Relevant simulation parameters have been scaled by the decay rate $\gamma$ where we denote $\gamma =\gamma _{1}=\gamma _{2}$. Unless mentioned differently or being explicitly indicated in the plots, these are the common values to all the plots presented below (it is also noted in the figure captions). As our study focuses primarily on the absorption and gain properties, we are interested in the imaginary part of $\rho _{31}^{(1)}$. Note, that although not demonstrated, in most cases the plots for the real part of $\rho _{31}^{(1)}$ (representing the dispersion) qualitatively look similar to the absoprtion ones, except for being rotated relative to them by some azimuthal angle, as well as having a different scale.

### Azimuthally dependent absorption and gain

#### Dependence on the OAM number

**Fig. 2:**
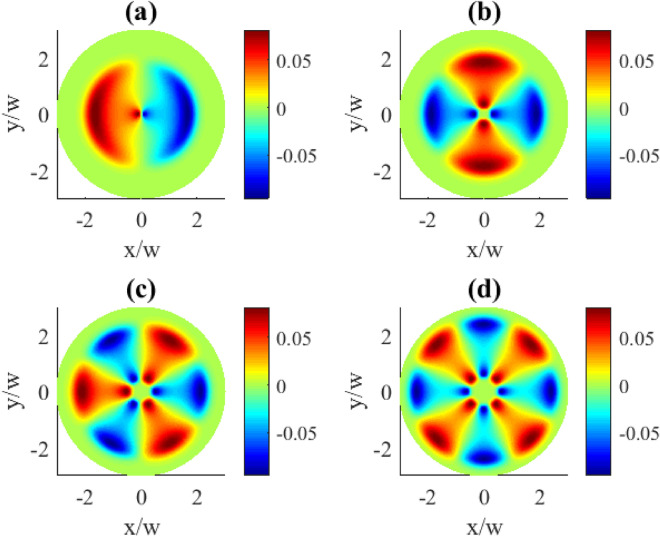
Transverse distributions of the imaginary part of $\rho _{31}^{(1)}(x,y)$ for different OAM numbers *l*: (**a**) $l=1$, (**b**) $l=2$, (**c**) $l=3$, (**d**) $l=4$. Other parameters: $r=0.1\gamma$, $\varOmega _{p0}=0.1\gamma$, $\varOmega _{c0}=10\gamma$, $\Delta _{p}=0$, $\Delta _{c}=0$, $\gamma _{1}=\gamma _{2}=\gamma$, $\eta =1$, $\varTheta =\pi /4$.

In Fig. 2 the transverse distributions of the imaginary components of the first order coherence $\text {Im}\,{\rho }_{31}^{(1)}$ on the OAM number *l* are presented. One can see that the transverse distributions exhibit specific types of symmetry. First, the profiles are rotationally symmetric around the beam axis with the azimuthal symmetry angle $\Delta \varphi =2\pi /l$. Second, for the even *l* numbers there is also a reflection symmetry at some axial planes, and the number of planes equals to *l* (see plots (b) and (d)).

**Fig. 3:**
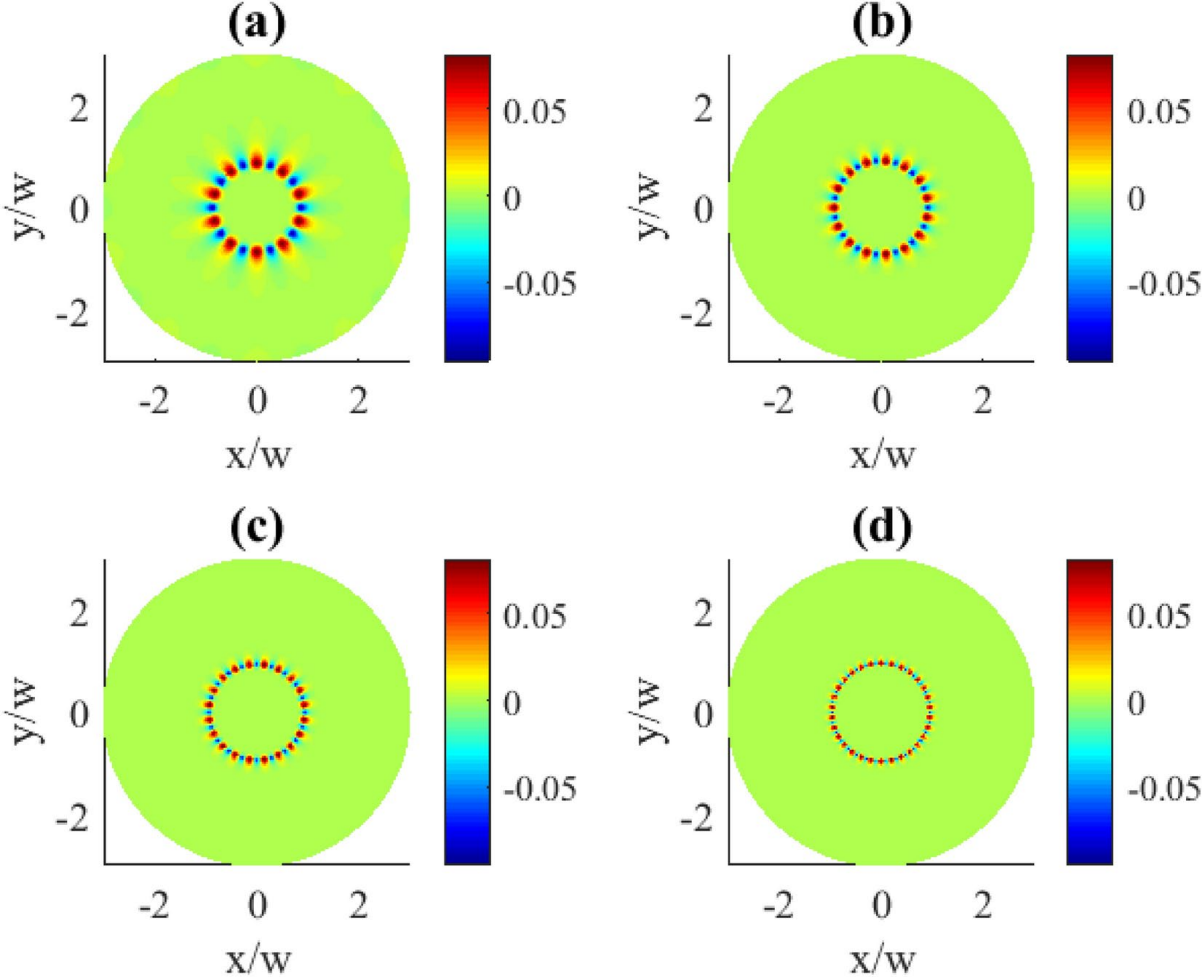
Transverse distributions of the imaginary part of $\rho _{31}^{(1)}(x,y)$ for different OAM numbers *l*: (**a**) $l=10$, (**b**) $l=15$, (**c**) $l=20$, (**d**) $l=30$. Other parameters: $r=0.1\gamma$, $\varOmega _{p0}=0.1\gamma$, $\varOmega _{c0}=10\gamma$, $\Delta _{p}=0$, $\Delta _{c}=0$, $\gamma _{1}=\gamma _{2}=\gamma$, $\eta =1$, $\varTheta =\pi /4$.

Note the periodic oscillations between the absorption ($\text {Im}\,\rho _{31}^{(1)}>0$) and gain ($\text {Im}\,\rho _{31}^{(1)}<0$) taking place along the azimuthal coordinate, seen in Fig. 2. With the increase of the OAM number the areas of absorption (red) or gain (blue) become squeezed and more localized ((c) and (d) plots) as a result of interference. A further increase in the localization of these regions happens when going to higher OAM numbers *l* (Fig. 3). Indeed, here we observe the collapse of the overall pattern into a well pronounced circular shape of the azimuthally modulated absorption and gain regions at the particular radial distance. The increase of the OAM number leads both to the compression in *R* and higher modulation frequency in $\varphi$ coordinates making stronger localization features.

#### Dependence on detuning

**Fig. 4:**
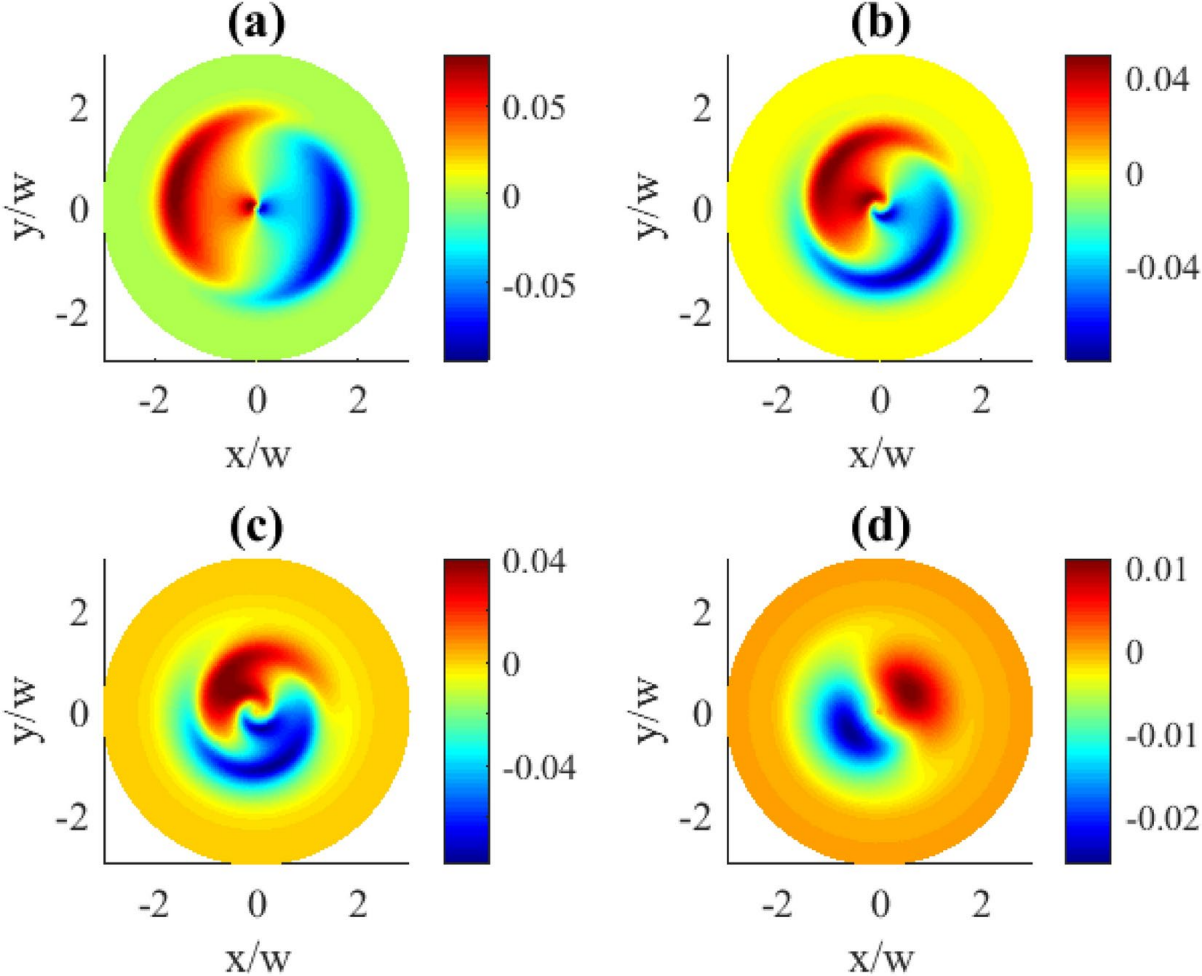
Transverse distributions of the imaginary part of $\rho _{31}^{(1)}(x,y)$ for different probe detunings $\Delta _{p}$: (**a**) $\Delta _{p}=0.1\gamma$, (**b**) $\Delta _{p}=1\gamma$, (**c**) $\Delta _{p}=2\gamma$, (**d**) $\Delta _{p}=5\gamma$. The OAM number $l=1$. Other parameters: $r=0.1\gamma$, $\varOmega _{p0}=0.1\gamma$, $\varOmega _{c0}=10\gamma$, $\Delta _{c}=0$, $\gamma _{1}=\gamma _{2}=\gamma$, $\eta =1$, $\varTheta =\pi /4$.

**Fig. 5:**
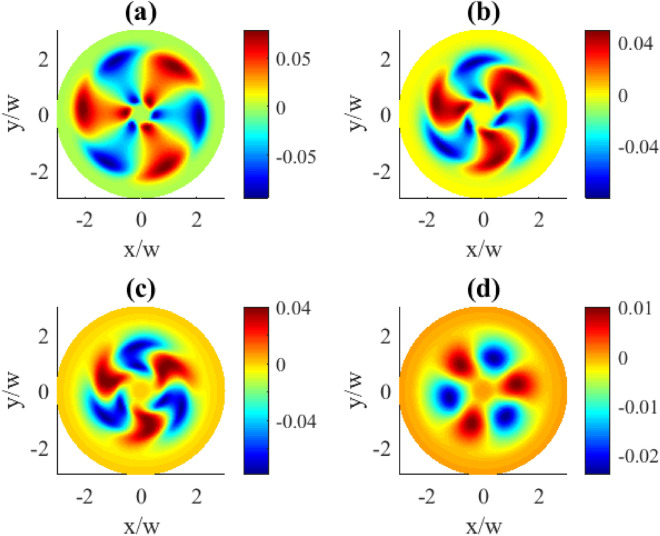
Transverse distributions of the imaginary part of $\rho _{31}^{(1)}(x,y)$ for different probe detunings $\Delta _{p}$: (**a**) $\Delta _{p}=0.1\gamma$, (**b**) $\Delta _{p}=1\gamma$, (**c**) $\Delta _{p}=2\gamma$, (**d**) $\Delta _{p}=5\gamma$. The OAM number $l=3$. Other parameters: $r=0.1\gamma$, $\varOmega _{p0}=0.1\gamma$, $\varOmega _{c0}=10\gamma$, $\Delta _{c}=0$, $\gamma _{1}=\gamma _{2}=\gamma$, $\eta =1$, $\varTheta =\pi /4$.

Controlling the detunings in the system can be achieved by changing either the probe detuning $\Delta _{p}$ or the control detuning $\Delta _{c}$. In Fig. 4 we demonstrate the effect by changing the detuning of the probe field $\Delta _{p}$. As expected, the detuning adds an extra phase variations to the coherence distributions leading to some peculiar profile deformations. Qualitatively this type of deformation can be described as the absorption/gain regions acquiring extended tails along the azimuthal coordinate what is mostly pronounced at the intermediate detunings (plots (b) and (c)). At larger detunings, however, this deformation becomes less visible, with the regions tending to be more rounded and localized (plot (d)). Tthe pattern dependencies on the probe detunings are presented for two OAM numbers $l=1$ and $l=3$ (Figs. 4, 5). Apparently, larger vorticities *l* lead to more complex and detailed transverse pattern shapes.

#### Dependence on other parameters

**Fig. 6:**
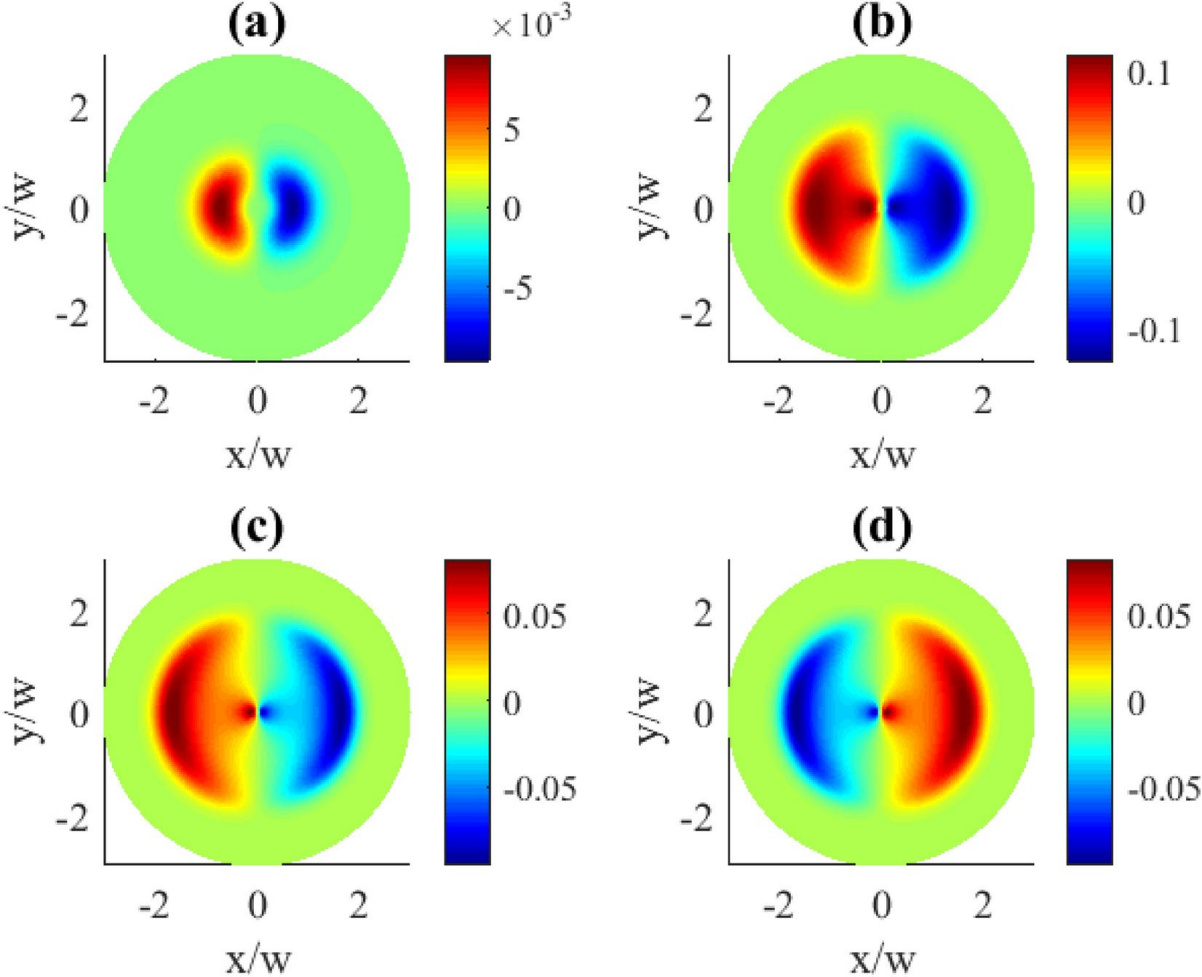
Transverse distributions of the imaginary part of $\rho _{31}^{(1)}(x,y)$ for different dipole angles $\varTheta$: (**a**) $\varTheta =\pi /100$, (**b**) $\varTheta =\pi /10$, (**c**) $\varTheta =\pi /4$, (**d**) $\varTheta =3\pi /4$. Other parameters: $r=0.1\gamma$, $\varOmega _{p0}=0.1\gamma$, $\varOmega _{c0}=10\gamma$, $l=1$, $\Delta _{p}=0$, $\Delta _{c}=0$, $\gamma _{1}=\gamma _{2}=\gamma$, $\eta =1$.

We explore the effect of the angle $\varTheta$ between the directions of the spontaneously emitting dipoles for $\varTheta$ in the range (0, $\pi$) excluding $\pi /2$ (shown in Fig. 6). For the very small $\varTheta$ values the patterns tend to be more localized towards the center of the beam (plot (a)) with a smaller amplitude. On the other hand, for the intermediate $\varTheta$ values it gets more extended in the radial direction retaining the azimuthal extent (plots (b) and (c)). Notably, comparing $\varTheta =\pi /4$ (c) and $\varTheta =3\pi /4$ (d) one can see the flip of the positive and negative regions, which can be attributed to $\cos \varTheta$ having different signs for $\varTheta <\pi /2$ and $\varTheta >\pi /2$.

**Fig. 7:**
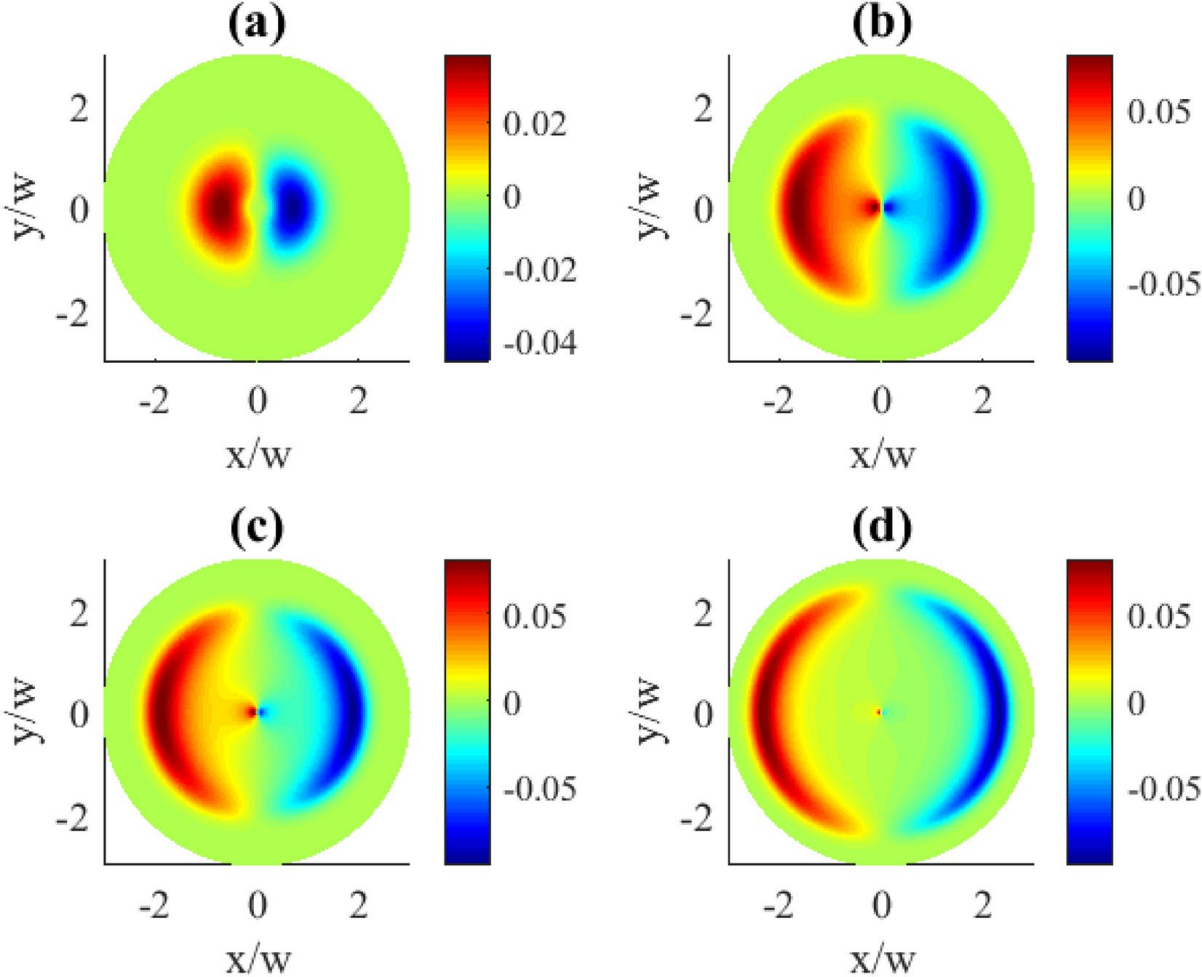
Transverse distributions of the imaginary part of $\rho _{31}^{(1)}(x,y)$ for different control field strengths (amplitudes) $\varOmega _{c0}$: (**a**) $\varOmega _{c0}=\gamma$, (**b**) $\varOmega _{c0}=10\gamma$, (**c**) $\varOmega _{c0}=20\gamma$, (**d**) $\varOmega _{c0}=100\gamma$. Other parameters: $r=0.1\gamma$, $\varOmega _{p0}=0.1\gamma$, $l=1$, $\Delta _{p}=0$, $\Delta _{c}=0$, $\gamma _{1}=\gamma _{2}=\gamma$, $\eta =1$, $\varTheta =\pi /4$.

Regarding the control of the vortex field amplitude $\varOmega _{c0}$ (see Fig.7), one can see that the effect of increasing $\varOmega _{c0}$ mostly leads to the shift of the corresponding patterns along the radial coordinate, while preserving the extent in the azimuthal coordinate. At high enough control field intensity (plot (d)), the absorption and gain regions become well separated in the radial direction.

**Fig. 8:**
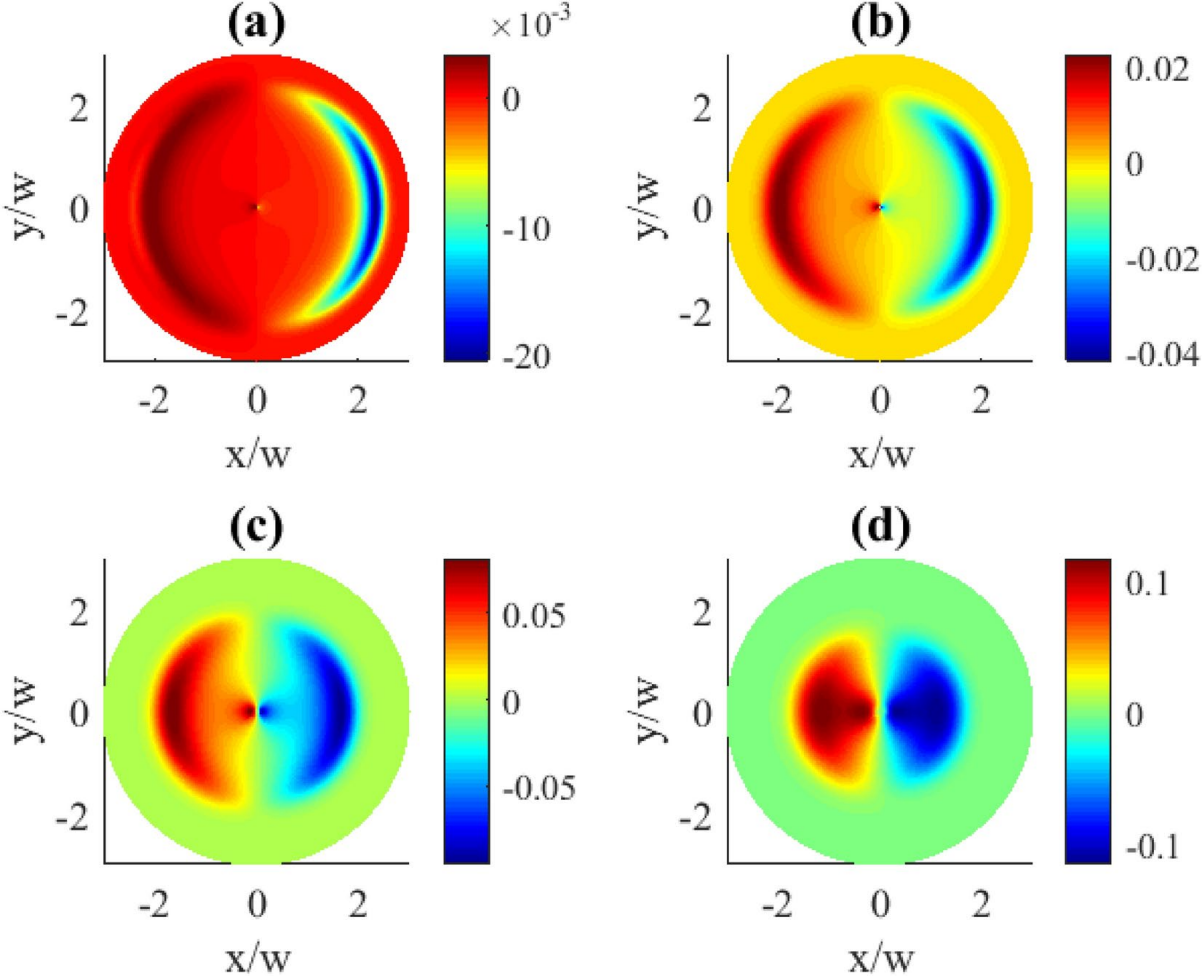
Transverse distributions of the imaginary part of $\rho _{31}^{(1)}(x,y)$ for different incoherent pump rates *r*: (**a**) $r=0.001\gamma$, (**b**) $r=0.01\gamma$, (**c**) $r=0.1\gamma$, (**d**) $r=\gamma$. Other parameters: $\varOmega _{p0}=0.1\gamma$, $\varOmega _{c0}=10\gamma$, $l=1$, $\Delta _{p}=0$, $\Delta _{c}=0$, $\gamma _{1}=\gamma _{2}=\gamma$, $\eta =1$, $\varTheta =\pi /4$.

Further, we analyzed the dependence on the incoherent pump rate *r* (Fig. 8). It mostly has the opposite effect compared to the previously studied on $\varOmega _{c0}$, as here the increase of *r* tends to squeeze the pattern towards the beam center (plot (d)). At small *r* values, the spatial dependence becomes less pronounced and weak. In the limit when $r=0$ there should be no spatial dependence, in accordance with our previous discussion on SGC.

### Azimuthally dependent upper state population

In this subsection we demonstrate the dependencies for the absolute values of the coherence $\rho _{11}$ related to the population of the upper state $|1\rangle$. This study shows that the population exhibits zero or weak azimuthal dependence on most of the relevant parameters, except for the OAM number.

#### Dependence on the OAM number

**Fig. 9:**
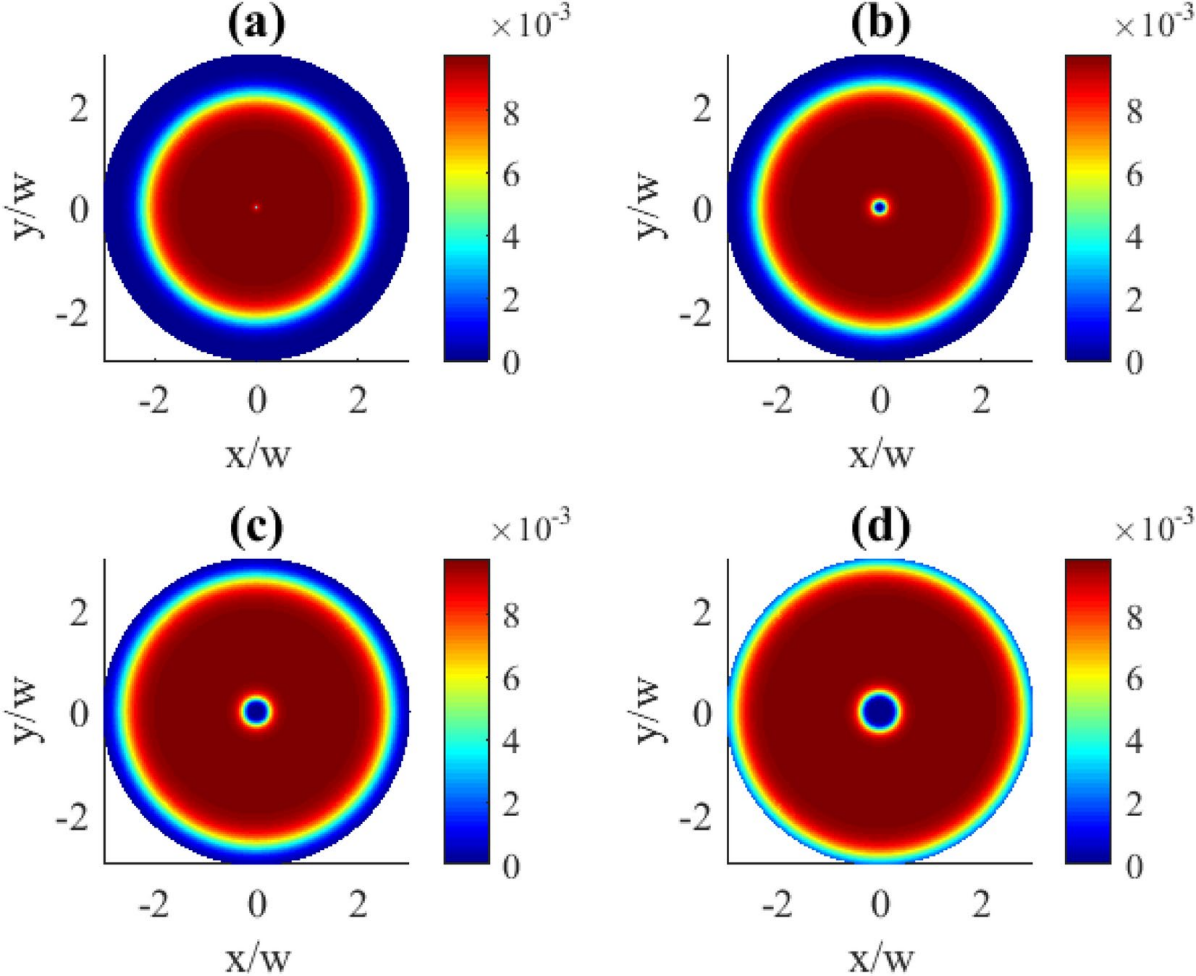
Transverse distributions of $|\rho _{11}^{(0)}(x,y)|$ for different OAM numbers *l*: (**a**) $l=1$, (**b**) $l=2$, (**c**) $l=3$, (**d**) $l=4$. The pump rate $r=0.01\gamma$. Other parameters: $\varOmega _{p0}=0.1\gamma$, $\varOmega _{c0}=10\gamma$, $\Delta _{p}=0$, $\Delta _{c}=0$, $\gamma _{1}=\gamma _{2}=\gamma$, $\eta =1$, $\varTheta =\pi /4$.

In order to give a better picture of the underlying effect, we first demonstrate the zero order population $\rho _{11}^{(0)}$ as dependent on the OAM number in Fig. 9. For the given OAM numbers of the vortex field the distributions are dominated by the uniformly populated upper state (dark red areas). Apparently, there is a lack of population at the center of the plots (blue areas) which can be explained by Eq. (7). Indeed, at the distance $R=0$ which is the vortex core, the control field $\varOmega _{c}=0$, thus also the population $\rho _{11}^{(0)}=0$. In this case, when the population of the excited state $|1\rangle$ goes to zero, the atom is trapped in a superposition of the states $|2\rangle$ and $|3\rangle$. Provided distributions exhibit perfect circular shape without azimuthal dependence, and with the increasing OAM the zero population area just expands.

**Fig. 10:**
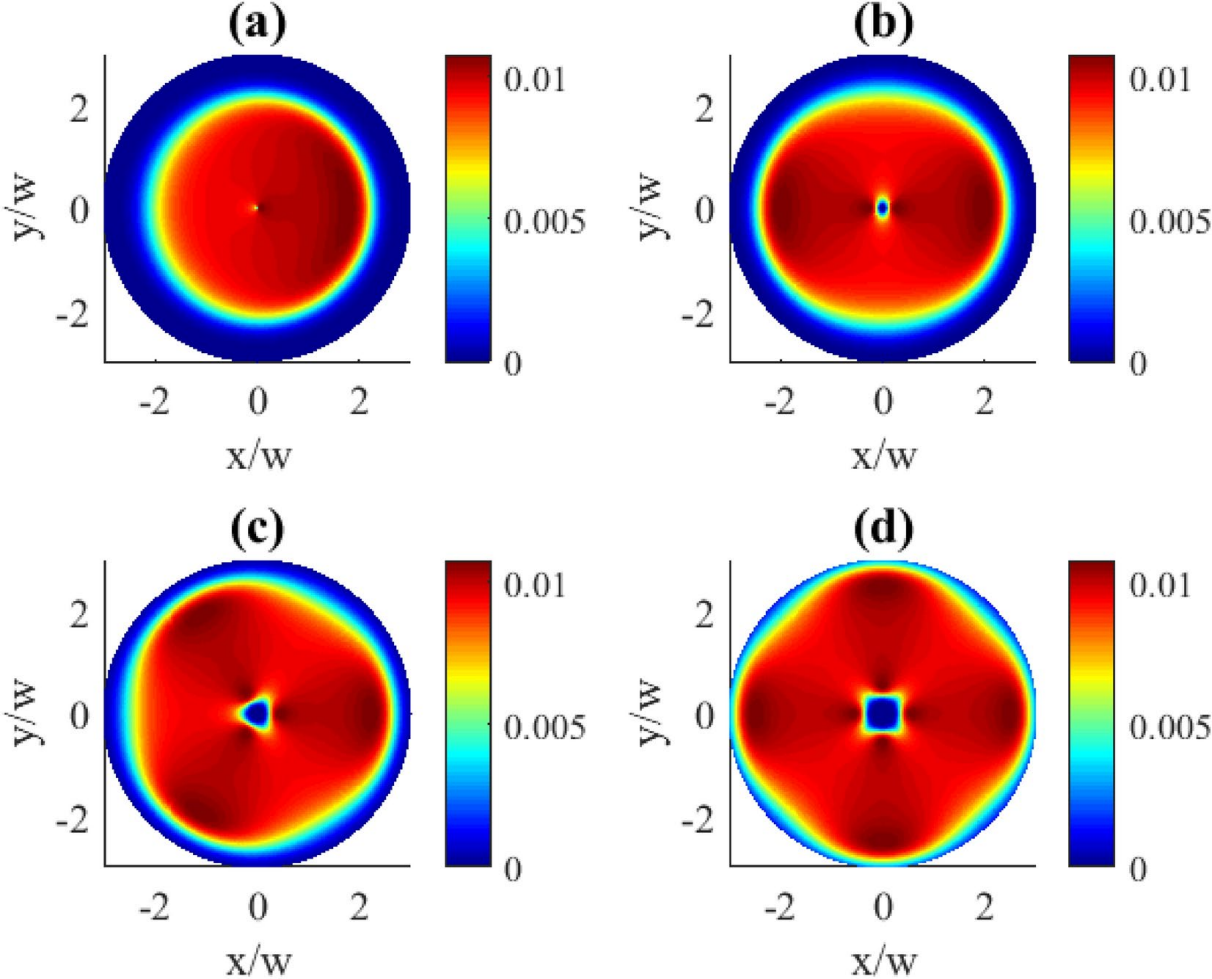
Transverse distributions of $|\rho _{11}^{(1)}(x,y)|$ for different OAM numbers *l*: (**a**) $l=1$, (**b**) $l=2$, (**c**) $l=3$, (**d**) $l=4$. The pump rate $r=0.01\gamma$. Other parameters: $\varOmega _{p0}=0.1\gamma$, $\varOmega _{c0}=10\gamma$, $\Delta _{p}=0$, $\Delta _{c}=0$, $\gamma _{1}=\gamma _{2}=\gamma$, $\eta =1$, $\varTheta =\pi /4$.

Contrary, when we consider the transverse distributions of the first order population $\rho _{11}^{(1)}$ presented in Fig. 10, one can observe the appearance of the azimuthally dependent features. The central part which lacks population changes here from a circle to oval, then to triangle and square for the OAM numbers 1, 2, 3, 4, correspondingly, clearly indicating an azimuthal dependence. Along with that, the characteristic OAM-induced petals in the population patterns can be seen in the (b), (c), and (d) plots. The demonstrated population distributions exhibit the same types of symmetries as previously described for the absorption plots in Fig. 2.

## Discussion

The alternating gain and absorption patterns is a direct consequence of the structured phase profile of the vortex control beam. The helical phase front associated with OAM beams imposes an azimuthal variation in the coherence properties of the system, which in turn modulates the optical response. The presence of SGC further enhances this effect by enabling quantum interference between different decay pathways. Manipulating the angle $\varTheta$ changes the strength of SGC, resulting in a modulation of the azimuthal distribution of absorption/gain properties. From Eq. (15), it follows that in certain azimuthal regions, the imaginary part of $\rho _{31}^{(1)}$ becomes negative, corresponding to gain, while in others, it becomes positive, leading to absorption. As the OAM number $l$ increases, the azimuthal phase gradient steepens, causing the phase to vary more rapidly along $\varphi$. Since SGC influences the coherence between ground-state levels, the optical response is sensitive to these variations. The control beam modulates the coherence properties, and increasing *l* enhances this azimuthal modulation due to the rapidly changing phase of the vortex beam. The transverse optical response (gain and absorption) results from interference between the transitions driven by the probe and control beams. Higher *l* values introduce finer azimuthal phase variations, creating regions where constructive and destructive interference become more confined. This leads to the observed squeezing of absorption and gain areas, with their widths decreasing as *l* increases. Beyond azimuthal localization, increasing *l* also affects the radial intensity profile of the vortex beam. For higher *l*, the intensity ring of the vortex control field shifts outward, modifying the region where the strongest coherence effects occur. As a result, the overall pattern of absorption and gain becomes more compressed radially while maintaining a high modulation frequency in the azimuthal direction.

In general, the key control parameters of the system - such as the incoherent pump rate, probe detuning, and control field strength - each play a crucial role in shaping its coherence properties by modifying the interaction dynamics between the atomic medium and the applied fields. The strength of the control field determines the degree of coherence coupling, shifting the spatial distribution of absorption and gain regions. Probe detuning alters the resonance conditions between the atomic transitions and the applied fields, modifying the interaction strength and, consequently, the absorption and gain characteristics. The incoherent pump rate influences the population distribution, affecting the contrast and localization of coherence effects. Together, these parameters provide a means to control and tailor the optical response of the system.

## Conclusions

In summary, we studied the spatial patterns of absorption and gain in a 3-level $\Lambda$-system with spontaneously generated coherence (SGC) and incoherent pump where the control field is an optical vortex. Due to the inherent inhomogeneity of the vortex field both in its amplitude and phase, variable conditions are achieved across the whole transverse plane leading to azimuthal dependencies of the optical response on a number of relevant system parameters. Our study shows that the azimuthal dependency of the absorption and gain distributions can be controlled essentially by the orbital angular momentum (OAM) and detuning, while being weakly affected by the other parameters, such as the dipole angle, control vortex field strength, and incoherenct pump rate. The upper state population’s azimuthal dependence is mostly affected by the OAM number, but shows no or weak changes for the other parameters. It should be noted that the absorption and gain profiles are mostly dominated by the zero-order coherence contribution. This occurs because the latter already incorporates the azimuthal modulation induced by the effects of control field, incoherent pumping, and SGC. The resulting structure of the shown first-order coherence is weakly influenced by the probe field, making rather small contribution to the total coherence.

The presented approach provides high flexibility in handling and patterning the optical response characteristics, and as such, contributing to advancing optical information processing, communication, and sensing. The ability to manipulate the spatial distribution of absorption and dispersion using structured light, such as vortex beams carrying OAM, introduces additional degrees of freedom for controlling light-matter interactions. This spatially dependent modulation can be particularly useful in quantum sensing applications, where precise control over optical properties enhances sensitivity to external perturbations. Furthermore, the structured optical fields have been explored for quantum information processing, and our findings suggest that such coherence-driven control mechanisms could enable novel approaches for encoding and manipulating quantum states. The interplay between SGC and structured beams in our system also has potential implications for tailored light-matter interactions in non-classical light generation and high-resolution imaging techniques. These results highlight the broader applicability of our approach in emerging quantum technologies.

The results presented here can be affected by the temperature of the system, as higher temperatures enhance the dephasing through the thermal effects, thus modifying the effective decay rates, shortening coherence lifetimes, and weakening SGC. Indeed, the increased dephasing suppresses interference effects between the decay channels, potentially diminishing or eliminating SGC-induced modulation of the absorption and gain. In this regard, our model assumes a regime where the coherence effects are significant, such as observed in cold atomic gases or cryogenically cooled solid-state systems. Under these conditions, the dephasing is minimized, allowing SGC to dominate the optical response.

While SGC has been experimentally observed in a V-type atomic system, such as sodium dimer^44^, where two closely spaced upper levels decay to a common ground state, realizing SGC in a $\Lambda$-type atomic system has remained challenging. This is due to the stringent requirement of simultaneously fulfilling the condition of the near degeneracy of the two lower levels and the non-orthogonality of the dipole moment transitions, which is difficult to achieve in such atomic systems. However, these conditions can emerge in more complex physical systems. For instance, an entangled state between a single ion and two modes of the radiation field has been demonstrated, an effect that is conceptually complementary to SGC^45^. Furthermore, experimental evidence suggests that SGC plays a significant role in certain condensed-matter systems, particularly in charged GaAs quantum dots, where an analogous level structure exists^46^. In the absence of an external magnetic field, the Zeeman sublevels of the conduction-band electron in a charged quantum dot remain degenerate. Upon optical excitation, the system forms a trion state consisting of a singlet electron pair and a heavy hole. When a magnetic field is applied in the Voigt geometry, the level structure transforms into an effective three-level $\Lambda$ system. Given that the Zeeman splitting in this scenario is comparable to or smaller than the trion decay rate, and the transition dipole moments to the two spin states are non-orthogonal, the necessary conditions for SGC are met. This suggests that quantum dots provide a promising platform for experimental realizations of SGC.

## Data Availability

The datasets used and/or analyzed during the current study are available from the corresponding author on reasonable request.
